# Temporal Features of Spike Trains in the Moth Antennal Lobe Revealed by a Comparative Time-Frequency Analysis

**DOI:** 10.1371/journal.pone.0084037

**Published:** 2014-01-20

**Authors:** Alberto Capurro, Fabiano Baroni, Linda S. Kuebler, Zsolt Kárpáti, Teun Dekker, Hong Lei, Bill S. Hansson, Timothy C. Pearce, Shannon B. Olsson

**Affiliations:** 1 Department of Engineering, University of Leicester, Leicester, United Kingdom; 2 School of Psychology and Psychiatry, Faculty of Medicine, Nursing and Health Sciences, Monash University, Clayton, Victoria, Australia; 3 NeuroEngineering Laboratory, Department of Electrical & Electronic Engineering, University of Melbourne, Melbourne, Victoria, Australia; 4 Centre for Neural Engineering, University of Melbourne, Melbourne, Victoria, Australia; 5 Department of Evolutionary Neuroethology, Max Planck Institute for Chemical Ecology, Jena, Germany; 6 Department of Zoology, Plant Protection Institute, Centre for Agricultural Research, Hungarian Academy of Sciences, Budapest, Hungary; 7 Division of Chemical Ecology, Swedish University of Agricultural Sciences, Alnarp, Sweden; 8 Department of Neuroscience, School of Mind, Brain and Behavior, University of Arizona, Tucson, Arizona, United States of America; University of California, Los Angeles, United States of America

## Abstract

The discrimination of complex sensory stimuli in a noisy environment is an immense computational task. Sensory systems often encode stimulus features in a spatiotemporal fashion through the complex firing patterns of individual neurons. To identify these temporal features, we have developed an analysis that allows the comparison of statistically significant features of spike trains localized over multiple scales of time-frequency resolution. Our approach provides an original way to utilize the discrete wavelet transform to process instantaneous rate functions derived from spike trains, and select relevant wavelet coefficients through statistical analysis. Our method uncovered localized features within olfactory projection neuron (PN) responses in the moth antennal lobe coding for the presence of an odor mixture and the concentration of single component odorants, but not for compound identities. We found that odor mixtures evoked earlier responses in biphasic response type PNs compared to single components, which led to differences in the instantaneous firing rate functions with their signal power spread across multiple frequency bands (ranging from 0 to 45.71 Hz) during a time window immediately preceding behavioral response latencies observed in insects. Odor concentrations were coded in excited response type PNs both in low frequency band differences (2.86 to 5.71 Hz) during the stimulus and in the odor trace after stimulus offset in low (0 to 2.86 Hz) and high (22.86 to 45.71 Hz) frequency bands. These high frequency differences in both types of PNs could have particular relevance for recruiting cellular activity in higher brain centers such as mushroom body Kenyon cells. In contrast, neurons in the specialized pheromone-responsive area of the moth antennal lobe exhibited few stimulus-dependent differences in temporal response features. These results provide interesting insights on early insect olfactory processing and introduce a novel comparative approach for spike train analysis applicable to a variety of neuronal data sets.

## Introduction

The discrimination of complex stimuli in the noisy natural background is an immense computational task for any sensory system. For the olfactory system in particular, what we perceive as a single odor is generally composed of many different molecules creating an odor mixture. In addition, the odor molecules travel through the environment as discrete filaments in a turbulent and stochastic odor plume [1], [2]. Odor recognition therefore requires the simultaneous elucidation of the identity and complexity of molecular mixtures in specific ratios and concentrations, and at specific points in time. Comparative analyses across several invertebrate and vertebrate species suggest that complex stimuli are coded by sensory systems in a spatiotemporal fashion (for olfaction in particular see [3]), determined by where (spatial patterning), when (timing and synchronicity), and how much (intensity) neuronal activity occurs.

In addition to ensemble information, the firing rate of the individual neurons within sensory systems such as olfaction also provides information concerning the stimulus [4], [5]. In fact, neurons in the first olfactory neuropil of both invertebrates and vertebrates are known to exhibit complex temporal firing characteristics in response to odor stimuli that last for several hundred milliseconds after the stimulus has ended [6]. In insects, odor stimuli are encoded by these firing patterns in several ways (see [7] for recent review). First, a large body of studies have found that odors can be coded by differences in response amplitude across individual neurons (“fast rate coding” [7]). Odors can also be coded by response latency (“latency coding” [8]–). Finally, odors can be represented in the post-stimulus firing period (so-called “trace coding”) [12]–[14].

In a previous study of the moth antennal lobe (AL), the first olfactory synapse and insect analog to the olfactory bulb [15], we found that odor mixtures were coded by a latency code, while odor concentration was coded by increased firing rate [9]. However, using traditional spike-counting methods such as the mean firing rate, we were unable to localize specific time periods during the response where these differences occurred. Peri-stimulus time histograms (PSTH) derived from spike trains provide more temporal information, but they are restricted to the bin size set by the experimenter. Therefore, subtle neural patterns such as odor trace coding, may be overlooked, as suggested by Nawrot [7].

In order to identify specific temporal features of the neuronal response, we require a method that can allow us to compare different data sets and find statistically significant spike train features localized in time. The Discrete Wavelet Transform (DWT) is a type of time-frequency transformation of sampled data that is able to localize features in both time and frequency using basis functions called wavelets [16]–[18]. The main advantage of the DWT (and other techniques such as multitaper spectrotemporal analysis) over time-resolved Fourier transform is that the time domain is decomposed on multiple temporal scales, and thus low frequencies are examined over longer time periods than high frequencies, providing multiple scales of time-frequency resolution [19]. Wavelet-based applications are not new to neuroscience [19]–[22], but their use for spike train analysis has been relatively scarce, even if some successful algorithms have been developed in the context of real-time spike train decoding for neural prosthesis [23], [24].

In this study, we calculate the DWT of the instantaneous firing rate function in order to localize temporal features relevant to discriminating different stimulus categories (i.e. different compounds, concentrations, and mixtures). We apply this multi-resolution time-frequency analysis to three different data sets, comprising neuronal responses recorded in sexually isomorphic glomeruli of female *Manduca sexta* [9] and in the pheromone-responsive macroglomerular complex (MGC) of male *Manduca* [25] and *Ostrinia nubilalis* [26]. In contrast to sexually isomorphic AL neurons that receive input from receptor neurons with a variety of tuning properties, macroglomerular neurons receive sensory input from highly specific receptor neurons responding to pheromone components. The question remains to what extent the specialized MGC system differs from the larger, sexually isomorphic antennal lobe portion [27]. The MGC is generally considered to be a labeled-line network with highly specific input, while the sexually isomorphic neurons utilize combinatorial processing with a mixture of specific and broadly-tuned OSNs [28], [29].

Previous analyses of these data sets used mean rate, PSTH, and latency measurement [9], [25], [26], and thus provide a suitable reference for assessing the additional insight provided by the DWT analysis. In addition, an interspecies comparison can reveal general physiological properties that could be widely applicable to several species, given the widespread sexual dimorphism of the AL among moths and other insects [30]. Hence, we apply our multi-resolution time-frequency analysis to assess how AL neurons from two separate sections of the moth antennal lobe provide information concerning odor identity, concentration, and presence of a mixture. In addition, we compare the time localization of spike train features obtained with our DWT analysis to a PSTH-based analysis. Finally, we discuss significant response features localized in time and frequency revealed by our application of the DWT, and its implications for understanding the mechanistic basis of odor coding in the antennal lobe.

## Materials and Methods

### Neuronal recordings and odor stimulation

**Figure 1:**
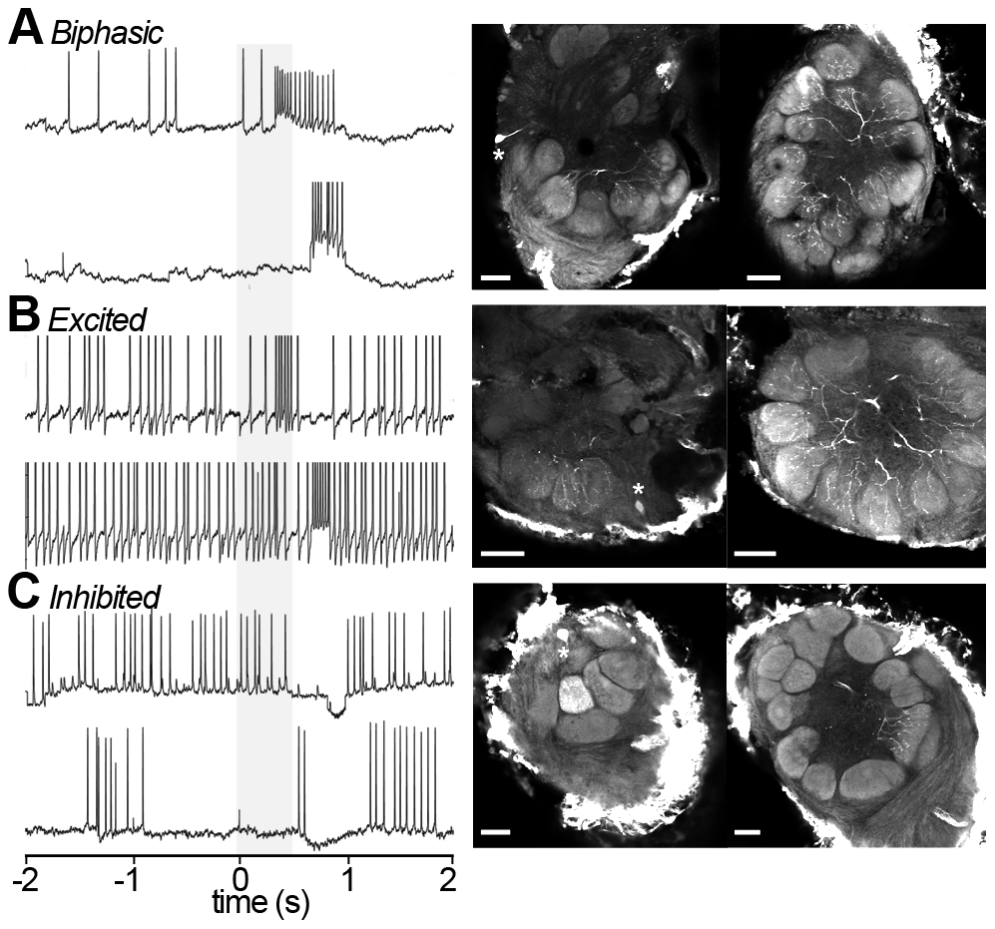
Basic temporal response types. (A) A biphasic neuron from *M. sexta* stimulated with a mixture (upper panel) and a single component at the mixture concentration (lower panel). (B) An excited neuron stimulated at low (upper panel) and high (lower panel) concentrations of a compound. (C) An Inhibited neuron stimulated with low (upper panel) and high (lower panel) concentrations of a compound. Stimulus elapse shown in grey. The panels on the right show confocal micrographs of the three respective female *M. sexta* AL neurons, each extracting a single optical orthogonal slice (soma with asterisks, left images). Neurobiotin-injected cells were stained with Alexa-conjugated Streptavidin. Pictures were obtained by confocal microscopy of three separate whole mount brain preparations using a 10×, 0.45-NA objective lens (C-Apochromat, Zeiss). Optical sections (1024×1024 pixel) were taken at intervals of 0.8 µm. A and B display LNs, while C shows a multiglomerular PN; scale bar: 50 micrometers.

We present a DWT of rate functions derived from intracellular recordings of AL neurons reported previously for the moth *Manduca sexta* [9], [25] and *Ostrinia nubilalis* [26]. Data sets were obtained using plant [9] and pheromone [25], [26] compounds, respectively. Raw membrane potential data was obtained from these studies for the current analysis. Please refer to these studies for details of animal and stimulus preparation, electrophysiological recording, and morphological analysis. Single trial responses to monomolecular host plant odors or pheromones and their mixtures were separated into the three response classes as previously described [9] (Figure 1).

Briefly, female *Manduca sexta* moths were immobilized in modified Falcon tubes and the primary olfactory processing centers of the brain (the antennal lobes) surgically exposed. Once intracellular contact was established using a sharp glass microelectrode, the ipsilateral antenna was stimulated with (+) linalool, (−) linalool, phenyl acetaldehyde, benzaldehyde, hexanol, nonanal or trans- 2-hexenyl acetate (used instead of nonanal in some experiments), and cis-3-hexenyl acetate. Each odor was dissolved at 10^−4^ in mineral oil. Stimulations were performed at 500 ms duration using a novel multicomponent stimulus device that equilibrated all odor concentrations according to vapor pressure [31]. Neurons were first presented with all components simultaneously, then each of the seven odors separately. Odors that elicited a response were tested together as the “mixture”. Finally, the single components that elicited a response were again tested separately at the total mixture concentration. Neurons were identified as projection neurons or lateral interneurons by either morphological staining or physiological characterization via measurement of spike width (see [9]). In the former case, Lucifer or neurobiotin was injected iontophoretically after physiological characterization.

For assessment of macroglomerular neurons, male *Manduca sexta* were immobilized in plastic tubes and male *Ostrinia nubilalis* (Z race) were immobilized in plastic pipette tips and the antennal lobes surgically exposed. For *Manduca* MGC recordings, juxtacellular, rather than intracellular recording was performed. Sharpened borosilicate glass capillaries were used to measure extracellular activity of single MGC neurons in a manner similar to perforated-patch recording. 50 ms stimulations of (E)-10,(Z)-12-hexadecadienal (bombykal), the primary component of the conspecific female's sex pheromone, (E)-11,(Z)-13-pentadecadienal (C15, a chemically more stable mimic of another essential component of the sex pheromone), and their blend were presented at various concentrations (10 ng and 100 ng used for the current study). After recording, neurons were injected iontophoretically with Lucifer Yellow CH for morphological characterization.

For *Ostrinia* recording, odorants were diluted in redistilled n-hexane and applied on a filter paper inside a Pasteur pipette. Stimuli were presented as a 500 ms stimulation of (Z) or (E)-11-tetradecenyl acetate and their mixture at a range of concentrations (1 ng and 10 ng data used for our analyses). The purity of the odorants was verified using GC. After physiological characterization, neurons were injected iontophoretically with neurobiotin.

### Discrete wavelet transform of spike trains

We made use of the DWT here to localize in time and frequency relevant features of instantaneous rate functions derived from spike trains encoding odors among AL neurons. The temporal period over which we performed the DWT for recordings in the isomorphic AL of *Manduca* and the MGC of *Ostrinia* lasted 1.4 s, starting when the odorant reached the antenna (stimulus onset; determined empirically in [9]) and continuing for 900 ms after stimulus offset. As control, we also analyzed the 1.4 s preceding the stimulus. For juxtacellular PN recordings from the *Manduca* MGC, the temporal period lasted 1.4 s, starting when the odorant reached the antenna (including a 120 ms mechanical delay) and continuing for 1350 ms after stimulus offset.

**Figure 2:**
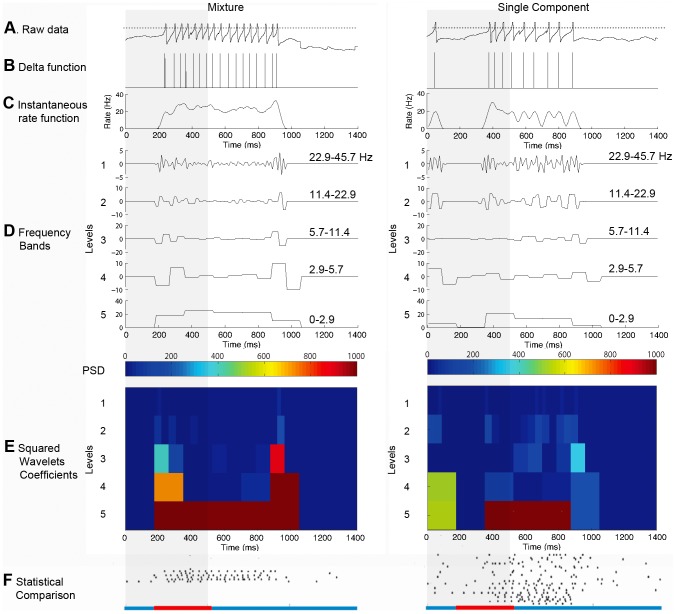
Example of DWT analysis for two traces. Left, response to a mixture, and right, response to a single component at mixture concentration. Stimulus period shown as gray bar. Raw traces (**A**) were transformed into sums of delta functions (**B**) from which instantaneous rate functions (in Hz) were determined. Dotted lines in (**A**) indicate set spike threshold. The rate functions were then separated into 128 bins (**C**) and used as input to the DWT, which decomposed the binned data into 4 frequency band levels 1–4 and one approximation (or scaling) level 5 (**D**). Color code indicates the power spectral density (PSD) corresponding to the resultant wavelet coefficients (**E**). The instantaneous spiking rate for each trace was thus decomposed in specific frequency bands and time windows. Finally, the corresponding PSD values (**E**) were statistically compared using a Mann-Whitney U test (complete data sets shown as rasters in **F**). The time windows associated with each wavelet coefficient were considered significant (red bars on the abscissa) when the uncorrected p values were smaller than the *crit_p* value obtained using a FDR level *q* set to 0.10 (Table 1).

The steps followed to obtain the wavelet coefficients for each trace are shown in Figure 2. First, spikes (Figure 2A) were detected when the membrane potential crossed a threshold set to half of the maximum peak amplitude. In the case of the juxtacellular *Manduca* MGC recordings the voltage was previously high pass filtered and the threshold was set to one third of the maximum peak amplitude. Detected spikes (Figure 2B) were then checked graphically to discard false positive or negative counts, and the threshold was varied in some cases to ensure a correct detection. The series of firing times for each trace were then represented as sums of delta functions (Figure 2B), which were convolved with unit area Hanning windows (half width = 50 ms) to obtain instantaneous rate functions expressed in Hz [32], [33]. It should be noted that the use of alternative Hanning window widths (e.g. 5 ms) did not significantly alter the overall results. The rate functions were divided into 128 bins (Figure 2C, bin duration = 10.94 ms) and decomposed into 4 frequency band levels (Figure 2D) using a dyadic discrete wavelets transform algorithm [17] with a Daubechies (db1) kernel [16]. In this way, we obtained 4 sets of detailed coefficients and 1 set of approximation coefficients that were squared to quantify the power spectral density (PSD) of the spiking rate function (plotted with color code in Figure 2E) in specific time windows and frequency bands. The number of detailed coefficients was as follows: 64 in the 1st level (22.86 to 45.71 Hz), 32 in the 2nd level (11.43 to 22.86 Hz), 16 in the 3rd level (5.71 to 11.43 Hz) and 8 in the 4th level (2.86 to 5.71 Hz). The approximation coefficients (0 to 2.86 Hz) were also 8. Thus, using the complete set of 128 coefficients, we constructed the frequency bands of the original signal. Following this procedure, the response and control periods were analyzed in successive time windows having different duration in each decomposition level, the duration being equal to the analyzed time (1.4 s) divided by the number of coefficients in the corresponding level.

The corresponding squared coefficients of the two data sets (e.g., responses to mixture and single component) were statistically compared (see Statistical Comparisons, below; Figure 2F) in order to localize significant features across the entire neuronal population. The pre-stimulus control periods were also compared for significant differences to control for false positives of the method. Ringing effects were minimized by the use of the db1 wavelet kernel, although we obtained similar results using other kernels (e.g. higher order db and rbio kernels).

### Statistical Comparisons

Using the DWT-based procedure described above, we searched for significant differences in the value of each squared coefficient for three comparisons: 1) mixture vs. single odorants at the mixture concentration, 2) single odorants at low vs. high concentration and 3) single odorants compared to each other. We divided the data set into subsets by response type (excited, inhibited, or biphasic neurons; Figure 1) and morphological type (projection neurons, PNs and lateral interneurons, LNs in *M. sexta*, and MGC neurons in *M. sexta* and *O. nubilalis*) following the classifications provided in [9]. Comparisons were performed only within these subsets. We assessed significant differences between correspondent squared coefficients with a Mann-Whitney U-test, using false discovery rate (FDR) [34]–[36] as a method to correct for multiple comparisons. Our null hypothesis was that there was no difference between two compared categories for any corresponding squared coefficients. The differences were considered significant when the uncorrected p values were smaller than the *crit_p* value obtained using a FDR level q set to 0.10 (Table 1). With this choice of q we did not find any significant difference in the pre-stimulus control periods of tested data sets. However, we also considered differences as marginally significant when the uncorrected p values were lower than a *crit_p* obtained using q<0.25 if the differences were found in the stimulus period and absent in the control period, and occurred during time periods not already indicated as significant (Table 1).

**Table 1:** Significant differences found between corresponding wavelet coefficients in the time-frequency analysis of biphasic and excited PNs from sexually isomorphic and MGC AL neurons of *Manduca sexta*.

Comparison^a^	Wavelet Level^b^	Wavelet Coefficient	StartingTime Window	EndingTime Window	Mann-Whitney U *p* value	FDR q value	FDR *crit_p* value
Biphasic Isomorphic PNs	1	10	196.875	218.75	0.0011	0.04	0.0022
Mixture vs.	1	11	218.75	240.625	0.0010		
single components	1	14	284.375	306.25	0.0016		
	2	5	175	218.75	0.0009		
	3	3	175	262.5	0.0016		
	4	2	175	350	0.0018		
	5	2	175	350	0.0013		
	1	9	175	196.875	0.0036	0.10	0.0091
	2	6	218.75	262.5	0.0043		
	2	7	262.5	306.25	0.0047		
	2	10	393.75	437.5	0.0022		
	3	4	262.5	350	0.0055		
	5	3	350	525	0.0084		
	1	58	1246.875	1268.750	0.0091	0.11	0.0123
Biphasic Isomorphic PNs	1	21	437.5	459.375	0.0079	0.13	0.008640569
Low vs. high	1	19	393.75	415.625	0.026	0.22	0.030016426
concentration	1	20	415.625	437.5	0.0086		
Excited Isomorphic PNs	1	52	1115.625	1137.5	0.0023	0.18	0.0028
Low vs. high	1	54	1159.375	1181.25	0.0104	0.22	0.0119
concentration	1	55	1181.25	1203.125	0.0070		
	4	2	175	350	0.0028		
	5	6	875	1050	0.0090		
	5	7	1050	1225	0.0080		
Excited MGC PNs	1	39	831.25	853.125	0.0027	0.22	0.0080
Mixture vs.	1	42	896.875	918.75	0.0027		
single components	3	13	1050	1137.5	0.0027		

^a^ All LNs, other MGC neurons and inhibited PNs exhibited no significant differences (see Results).

^b^ Wavelet levels correspond to the following frequency bands: level 1: 22.86 to 45.71 Hz, level 2: 11.43 to 22.86 Hz, level 3: 5.71 to 11.43 Hz, level 4: 2.86 to 5.71 Hz, level 5 (scaling): 0 to 2.86 Hz.

**Figure 3:**
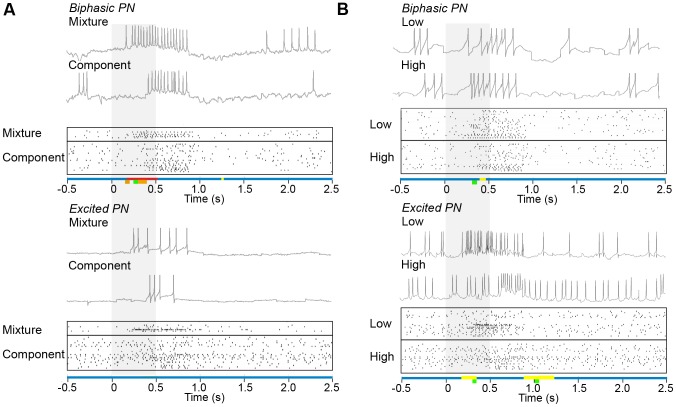
Temporal Response Patterns in sexually isomorphic AL neurons. (**A**) Raster plots showing the response of PNs to a mixture of 2–7 components and the single odorants at mixture concentration for biphasic (top) and excited (bottom) response types. (**B**) Raster plots showing the response of PNs to single odorants at low (1×10^−4^) vs. high (2–7×10^−4^) concentration as displayed as in **A**. Rasters include every recording from all sampled PNs for a given stimulus and response type. Each raster provides an example case on top showing the membrane potential recorded from same PN for the two different types of stimuli compared. Top row of colored bars on the abscissas indicate time windows where significant or marginally significant DWT differences in the temporal response patterns were found (red, FDR≤0.10; yellow, FDR<0.25, Table 1). In the second row of colored bars, time windows exhibiting significant differences via PSTH analysis are shown (orange, FDR≤0.10; green, FDR<0.25; note that FDR = 0.28 for comparison in **B** lower panel, Table 2). Stimulus timing is shown as a gray vertical bar.

The associated time windows are indicated with red bars (q≤0.10) or yellow bars (q<0.25) on the abscissas (Figures 2 and 3). As an additional test, we randomly shuffled stimulus labels within each data subset separately. After this manipulation, no significant differences were observed, indicating that the results are unlikely to arise by chance. The data analysis was performed using customized Matlab codes (File S1).

### Comparison to peristimulus time histogram analysis (PSTH)

**Figure 4:**
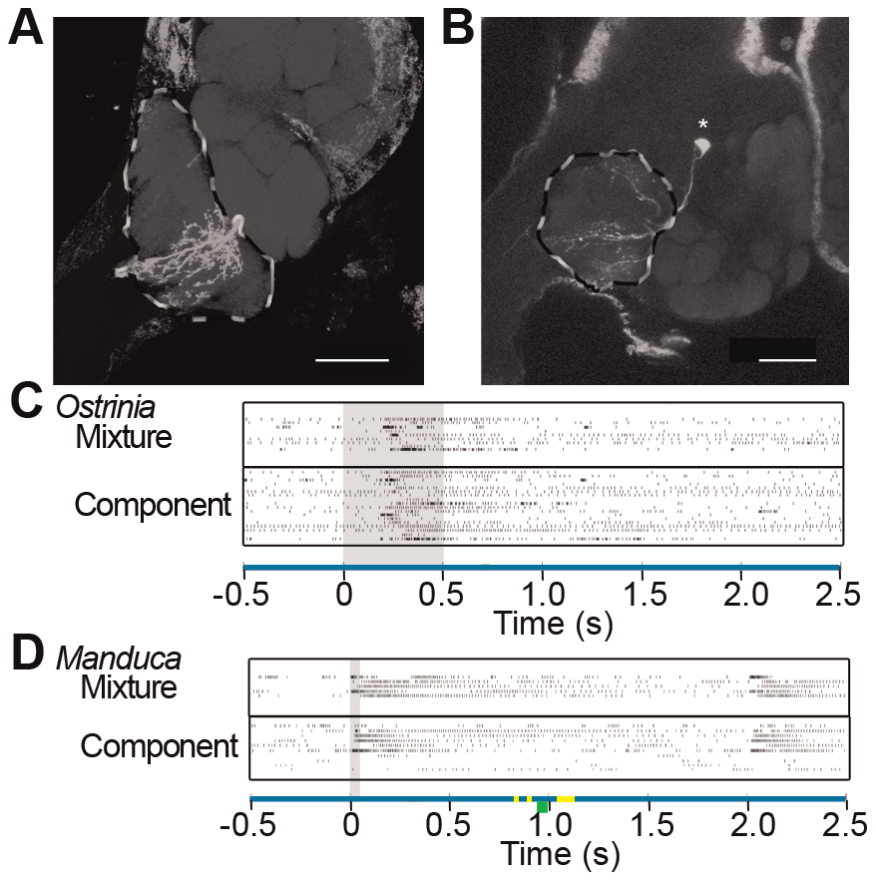
Temporal Response Patterns in the MGC. **A–B** Confocal micrographs of two male ALs of Z-strain *O. nubilalis*, each extracting an overlay of several optical orthogonal slices (38 in A, 20 in B). Neurobiotin-injected cells were stained with Alexa-conjugated Streptavidin and alpha-synapsin/Alexa for background staining. Pictures were obtained by confocal microscopy of two separate whole mount brain preparations using a 40×, 1.3 Oil DIC objective lens (Plan-Neufluar, Zeiss). Optical sections (1031/1024×1024 pixel) were taken at intervals of 0.9 µm (**A**) and 0.7 µm (**B**). **A** and **B** display PNs innervating the MGC (outlined with dotted line); soma in **B** indicated with asterisk; scale bar: 50 micrometers. **C–D** Raster plots showing spiking times in different MGC neurons with excited response type. (**C**) Raster plots of excited neurons in the *Ostrinia nubilalis* MGC stimulated with the mixture (upper panel; 10 ng total) and the individual pheromone components at the mixture concentration (lower panel). (**D**) Excited PNs in the *Manduca sexta* MGC stimulated with the mixture (upper panel; 100 ng loading) and the individual pheromone components at the mixture concentration (lower panel). Colored bars indicate significant differences as in Figure 3. Stimulus timing shown in grey.

The isomorphic AL neuron recordings of *Manduca sexta* that we studied here with DWT were previously analyzed using the mean firing rate and the area bellow the PSTH over the total response period (1.4 s) [9]. In order to compare the time localization obtained using the DWT with a standard temporal spike train analysis, we calculated PSTHs of the response and pre-stimulus control periods using a bin of 50 ms as in [9]. The spike counts found for each of the 28 bins were compared between the different conditions, in a similar manner as for the wavelet coefficients. The time periods found to be significant (Table 2) are shown with orange (q≤0.10) and green (q<0.25) bars under the abscissas of Figures 3 and 4. Thus, the red and orange bars compare significant windows for DWT and PSTH, while yellow and green bars compare marginally significant windows for both analyses. In the case of PSTH we included one case of q = 0.28 (*Manduca* isomorphic PNs, low vs. high concentration of components, green bars in Figure 3B bottom; Table 2) for the sake of comparison with DWT.

**Table 2:** Significant differences found in the PSTH analysis of biphasic and excited PNs from sexually isomorphic and MGC AL neurons of *Manduca sexta*.

Comparison^a^	Bin	StartingTime Window	EndingTime Window	Mann-Whitney U *p* value	FDR q value	FDR *crit_p* value
Biphasic Isomorphic PNs	7	300	350	0.0013	0.03	0.0016
Mixture vs.	4	150	200	0.0093	0.07	0.0096
single components	8	350	400	0.0016		
	6	250	300	0.0096	0.16	0.0273
Biphasic Isomorphic PNs	7	300	350	0.0078	0.24	0.0168
Low vs. high						
concentration						
Excited Isomorphic PNs	7	300	350	0.0239	0.28	0.0297
Low vs. high	21	1000	1050	0.0093		
concentration						
Excited MGC PNs	20	950	1000	0.0120	0.20	0.0140
Mixture vs.						
single components						

^a^ All LNs, other MGC neurons and inhibited PNs exhibited no significant differences (see Results).

## Results

We analyzed the response of 29 neurons (7 biphasic, 5 excited, and 9 inhibited PNs; 2 biphasic, 4 excited, and 2 inhibited LNs) in the sexually isomorphic glomerular portion of the *Manduca sexta* AL responding to low/high concentrations and mixtures of active plant volatiles [(+) linalool, (−) linalool, phenyl acetaldehyde, benzaldehyde, hexanol, nonanal or trans- 2-hexenyl acetate (used instead of nonanal in some experiments), and cis-3-hexenyl acetate]. We also analyzed 24 neurons (8 biphasic, 9 excited and 7 inhibited) in the MGC of *Ostrinia nubilalis* responding to low/high concentrations and mixtures of (*E*) and (*Z*)-tetradecenyl acetate, and another 5 PNs from the MGC of *M. sexta* responding to low/high concentrations and mixtures of Bombykal and C15. Within each morphological and response type, we compared the response of the neurons to single odorants, to single odorants at low vs. high concentrations and to mixtures vs. single odorants at the mixture concentration. In Figure 3 we present the cases in which significant differences were found in the time-frequency analysis of the instantaneous rate function in sexually isomorphic AL neurons of *Manduca sexta*. The results for MGC neurons of *Ostrinia nubilalis* and *Manduca sexta* with excited response profiles are depicted in Figure 4.

PNs showing biphasic response in sexually isomorphic AL neurons of *Manduca sexta* displayed higher firing rate for mixtures than for single components at the mixture concentration in a long lasting time window from 175 ms to 525 ms after stimulus onset (Table 1; Figure 3). This suggests that biphasic PNs respond to stimulus mixtures with a shorter latency than to single components. The difference was localized across all frequency bands (levels 1–5; 0 to 45.71 Hz) early in the response onset (175 to 350 ms). In the low frequency band (level 5, 0 to 2.86 Hz, Table 1) of the instantaneous rate function, the difference extended from 175 to 525 ms. Thus, no significant differences were observed 25 ms after stimulus offset, indicating that the larger response evoked by the mixture occurred only within the stimulus period. These biphasic isomorphic PNs also exhibited a marginally significant difference in response to different concentrations of single odorants (Figure 3B, top) occurring in the highest frequency band (level 1, 22.86–45.71 Hz) during a small 66 ms time window from 394–459 ms following stimulus onset. The significant wavelet coefficients are listed in Table 1.

PNs showing excited responses in the sexually isomorphic portion of the *Manduca sexta* AL exhibited marginally significant differences in response to different concentrations of single odorants (Figure 3B, bottom). In a early time window (175 to 350 ms), the lower concentrations evoked larger response than the higher concentrations only in the wavelet level 4 corresponding to the frequency band 2.86 to 5.71 Hz. Conversely, the higher concentrations evoked larger responses in a late time window from 875 to 1225 ms after stimulus onset localized in 2 different frequency bands, corresponding to wavelet levels 5 (0–2.86 Hz) and also 1 (22.86–45.71 Hz) during part of this time period (see Table 1). This indicates that higher concentrations of individual odorants evoked responses that were less intense during the stimulus, but the spike trains were sustained at an increased rate for longer time after stimulus offset (at least 350–700 ms following the stimulus).

No other significant windows were found in PNs or LNs of different response profiles in *Manduca sexta* for mixtures, concentration or identity of single components (see examples in Figure 3). We also obtained no significant differences among neurons responding to pheromone components in the MGC of *Ostrinia nubilalis* (Figure 4), suggesting that the individual firing patterns observed in the responses do not provide significant temporal information to discriminate compounds, concentrations, or mixtures.

To confirm whether the observed differences were a result of AL portion (isomorphic glomeruli or MGC), species (*Manduca* or *Ostrinia*), or morphological type (i.e. projection or inter-neuron), we additionally assessed the response of MGC PNs of *Manduca sexta* (Figure 4). In this data set, all PNs exhibited excited responses. Marginally significant differences were found in a series of discrete time windows roughly 800 ms following stimulus offset (Table 1; Figure 4). These responses were confined to wavelet levels 1 (22.86–45.71 Hz) and 3 (5.71 to 11.43 Hz) in discontinuous windows between 831 and 1137 ms following stimulus onset. As a result, it appears that the differences observed in *M. sexta* isomorphic PNs are predominantly a result of their innervation of isomorphic glomeruli in the AL, rather than their species origin (*Manduca*) or morphological type (PN).

The comparisons in which we found significant differences in the sexually isomorphic portion of the *Manduca sexta* AL (mixture vs single odorants in PNs with biphasic response and single odorants at high vs low concentrations in excited PNs) are in general agreement with the findings of [9]. The time localization obtained using DWT (Table 1) and PSTH (Table 2) occurred in similar time periods (compare red vs. orange and yellow vs. green bars in Figs. 3 and 4). However, the segments found significant with the DWT occurred over longer durations and in most cases reached lower FDR values than with the PSTH.

## Discussion

Here, we apply a new approach based upon the DWT to assess sexually isomorphic and specialized portions of the moth AL and statistically localize particular features of spike trains in both time and frequency that discriminate odor stimuli. As applied to existing data sets, the DWT analysis not only confirmed results obtained by rate and latency analyses [9], [26], but exposed novel information-bearing temporal features within the response train. Below, we discuss these differences in temporal patterning from sexually isomorphic and specialized neurons of the moth AL, and suggest potential sources for such localized response features.

### A “latency code” for mixtures in sexually isomorphic AL neurons

In biphasic PNs, the onset latency was significantly shorter for mixtures than for single components at the mixture concentration. It is important to note that, as discussed previously [9], these observed differences in latency are a product of neuronal processing of mixtures rather than stimulus presentation, as component and mixture intensities were equilibrated using a unique stimulus presentation system (for more information see [31]). In addition, these differences in latency were not observed when presenting different intensities of the individual components themselves (Figure 3). Thus, our results confirm the finding of the previous study [9] that mixture information is transmitted by the AL more quickly than for its components using a “latency code” [8], [9]. However, our previous study could not localize these differences in time and frequency. The present DWT analysis shows that the latency code is present from very low (<3 Hz) to relatively high (11–46 Hz) frequencies.

The observed differences in response between mixtures and components also end abruptly following stimulus offset. This suggests that any mixture information carried in the localized temporal response features does not persist after the stimulus has ended. Increased stimulus information during signal transients (onset and offset) has also been reported in PN responses in the locust [4]. In addition, a study in *Bombyx mori* [37] showed that onset and offset responses have different properties and might rely on different mechanisms. The source of this offset response might be due to peripheral input to the AL. Temporal coding of OSNs for identity, concentration, and stimulus timing has been shown to directly influence resultant PN responses [38]. A recent paper also suggests that PNs in the MGC of moths closely track the stimulus duration, and that this tracking could be due to inhibitory LN connections driven by OSN input [39].

The broad frequency band (0–45 Hz) of the differences in response to mixtures vs. single components is noteworthy in that it persists for only 175 ms in the middle of the stimulus window (ending at 350 ms after stimulus onset). Interestingly, this broad frequency information exists in a time window closely preceding the response time to conditioned odor found in insects (honeybees; ∼400 ms [40]). This broad frequency band including high frequency differences in response to mixtures may allow a more effective recruitment of Kenyon cells in the mushroom body ([41], see below). A similar time period was also observed for low frequency differences (2.86–5.71 Hz) between responses to different concentrations in excited PN cells (Table 1), with low concentrations evoking larger responses than higher concentrations (Figure 3B, bottom panel). However, our analyses were performed on purely physiological data and cannot directly confirm any connection to higher order processing, learning, or behavior. Nevertheless, these results suggest that our DWT analysis could provide an important tool to reveal unique spike information that could potentially be correlated to behavioral output.

### Long-term “odor trace coding” for concentrations in the moth AL

Single components presented at two different concentrations elicited specific changes in the slow temporal kinetics of excited type PNs in the sexually isomorphic neurons of the *M. sexta* AL (Figure 3, Table 1), but no significant differences in localized temporal response features were found in other isomorphic neurons. Although these values were marginally significant (Table 1, q = 0.22) due to the large variation within each dataset (Figure 3-B, bottom panel), they far exceeded any differences within the control period for these cells (q = 0.5). In addition, our previous analysis of the data [9] also found differences in response to concentration among excited type neurons, but could not localize these differences in time within the response. Using the DWT analysis, we discovered that the differences occurred in two different time periods, an early window (175 to 350 ms) in which low concentrations elicited larger response only at low frequencies (2.86–5.71 Hz), and a late window where the response was larger for high concentrations. This late window explains the results in [9], in which the response averaged along the total 1.4 s response time was found to be larger for higher concentrations. In this last window both low and high frequency differences (<2.86 Hz, 22.86–45.71 Hz; Table 1) were found late in the post-stimulus period (375–725 ms after stimulus offset), suggesting a form of “odor trace coding” [12]–[14] for concentration in the sexually isomorphic portion of the AL.

Recent trace conditioning versions of classic Pavlovian learning experiments in moths [42], bees [13] and flies [14] show learning even when the unconditioned stimulus is separated from the conditioned stimulus in time (i.e. there is no overlap in time between stimuli). However, these studies could find no correlation with trace conditioning and trace coding at either sensory or projection neuron levels. Intriguingly, the high frequency differences in response to concentration were only present in this later time window (22.86–45.71 Hz; ∼600–700 ms following stimulus offset), and correspond well with the hypothesized integration time constant (i.e. maximal separation of incoming EPSPs that still results in temporal summation) for Kenyon cells in the mushroom body (12–35 ms or 14–41 Hz, see Figure 2 in [41]), the proposed learning center of the insect brain [29]. This provides a tantalizing hypothesis that such high frequency information might provide higher brain centers with trace information for learning and memory. Although our data were derived from purely physiological analyses, the presence of long term differences in odor trace coding among excited projection neurons suggest that a similar use of our wavelets analysis on paired trace conditioning and physiological studies may reveal subtle differences in coding not seen using the traditional spike or calcium trace analyses.

### Localized temporal features in the MGC vs. sexually isomorphic AL neurons

Our assessment of neurons residing in the sexually isomorphic portion of the *M. sexta* AL and the pheromone-specific macroglomerular complex (s) of *Ostrinia nubilalis* suggests that localized temporal response features are neither *de facto* of, nor a necessity for, pheromone coding. When presented with single pheromone components at low and high concentrations as well as the mixture itself, no significant differences in localized temporal response features emerged for MGC neurons. To confirm that these results were not due to species-specific differences, we additionally assessed the response of MGC PNs in *Manduca sexta*. Few significant differences were found, including a series of discontinuous marginally significant differences roughly 800–1100 ms following stimulus onset at frequencies between 5.71–11.43 and 22.86–45.71 Hz. The sparse and discontinuous nature of the results for both MGC data sets in comparison to isomorphic *Manduca* PNs suggests that they do not likely play a significant role in odor coding, although the late time periods may suggest some role for trace coding (see above section). The apparent differences between temporal coding in sexually isomorphic vs. MGC neurons may reflect the specificity of their input, rather than any distinction in cellular properties or overall network connectivity. A “mixture response” in the sexually isomorphic portion of the AL reflects the activity of several OSNs [43] with different affinities and responses to odor components [44], [45], while the MGC response here results via input from only a few OSN types specific for pheromones (two in this study, [46], [47] and references therein). This creates a much higher level of OSN and lateral input within the sexually isomorphic neurons, which alters the slow temporal response. In addition, pheromone components can be coded as a labeled-line from OSN to behavior [48], [49], which does not impose, or necessitate complex temporal coding.

### DWT analysis of rate functions

Our application of the DWT to spike trains was inspired by its use in the processing of evoked potentials (e.g. [50]). As the spike firing times form a discontinuous signal, we used rate functions as the input signal for the DWT analysis. These rate functions were obtained from the spike trains with a symmetric convolution filter (Hanning window) to achieve a satisfactory localization in time (width can vary without significantly altering the results). The rate functions obtained (e.g. [33]) provide a relatively smooth representation of spiking activity, and minimize artifacts in the DWT coefficients due to the coarse quantization of binned spike-counts.

In the field of evoked potentials, a subset of wavelet coefficients is used for signal characterization. These relevant coefficients are often selected by fixing a threshold (e.g. [50]) or by picking the coefficients (manually or automatically) that prove to be useful for a specific purpose, for example to de-noise an evoked potential signal [19], [51]. In this context, our approach was to statistically compare the coefficients obtained for the response to different types of odor stimuli (e.g., mixture vs. single components, or low vs. high concentration) for all available neurons to determine which coefficients presented significant differences. We then highlighted the times associated with them in raster plots to discuss their biological relevance.

In comparison to the PSTH analysis of the spike data (compare Tables 1 and 2 and colored bars in Figures 3 and 4), the time localization of the differences that we found with DWT was similar, albeit over longer time periods and generally reaching lower FDR values. The difference between the two analysis methods could be due to the fact that the DWT can detect differences in several frequency levels, and the significant windows are formed by adding the times corresponding to all levels where significant differences are found, thus yielding greater sensitivity in comparison to a PSTH-based analysis at the same FDR level. Note that the main DWT differences often appear in several frequency levels (Table 1). The DWT also uses time windows of different duration for each wavelet level, while the PSTH-based analysis uses only a single bin duration. In addition to this localization in time, the DWT provides localization of differences in the frequency domain, which is not achieved by the binned spike counting. These multiple scales of time-frequency resolution constitute the main advantage of the DWT. In other words, while both the DWT and PSTH-based analyses reveal similar time periods where significant differences are observed, only the former method provides information regarding the frequency bands where these differences occur. This frequency information can enable additional inferences as to the nature of the neuronal processing, and in particular on the local or global scope of the information being processed. For example, information present in the DWT levels one and two (higher frequencies) is expected to be more efficiently conveyed to higher brain areas since it closely matches the time-scales of synaptic integration in the Kenyon cells of the mushroom body [41], while significant differences in higher levels (lower frequencies) might reflect mainly local processing.

### Future Directions

Our use of the DWT analysis on sexually isomorphic and specialized MGC neurons in the moth AL suggests that localized temporal responses at specific frequencies can lead to the emergence of olfactory “features” for higher order processing. Slow feature analysis [52], [53] provides a mathematical description for how slowly varying features can be coded from dynamic input, and may provide a novel approach to study the temporal responses of antennal lobe neurons. A recent study applied this analysis to simulated cortical microcircuits and found that slow pattern discrimination allows networks to extract information over several hundred ms and learn without supervision to code time relative to stimulus onset [54]. Although not yet applied to olfactory circuits, such a mechanism could explain the slowness of the antennal lobe as a principle for self organization [52] in the olfactory system. In line with these ideas, our analysis suggests that localized temporal features of spike trains are indeed playing a role in the encoding of mixtures and concentration levels in the sexually isomorphic AL neurons of *Manduca sexta*, though not in the MGC of *Ostrinia nubilalis* or *Manduca sexta*. Dynamical modeling techniques, as have been used recently to assess mixture processing in the sexually isomorphic [43] and MGC [28] AL networks, could potentially assess the role of slow feature analysis in olfactory processing.

## Supporting Information

File S1 **MATLAB m files for the calculation of DWT coefficients, statistical comparison and multiple comparison correction using FDR.** Compressed folder contains the following files: fdr_bh.m, batch_DWT.m, getDWTcoeff.m, DWT_spiketimes_analysis.m,, and example_data_DWT.mat. File “batch_DWT.m” is the main script. Example spike time data found in “example_data_DWT.mat”. When executed with the indicated parameters, “batch_DWT.m” outputs the results reported in Table 1 for a FDR of 0.1 using the example biphasic PNs of recorded from the isomorphic portion of the *Manduca sexta* antennal lobe. All scripts and functions are custom, with the exception of “fdr_bh.m”, which is also available from http://www.mathworks.com.au/matlabcentral/fileexchange/29274-mass-univariate-erp-toolbox/content/fdr_bh.m (last accessed 16/12/2013).
(ZIP)
