# Correction: Temporal Features of Spike Trains in the Moth Antennal Lobe Revealed by a Comparative Time-Frequency Analysis

**DOI:** 10.1371/journal.pone.0093026

**Published:** 2014-03-18

**Authors:** The PLOS ONE Staff

In the Author Contributions section, Hong Lei (HL) should be listed as one of the persons who contributed reagents/materials/analysis tools.
